# A reasonable Approach for the Treatment of HIV Infection in the Early Phase with Ozonetherapy (Autohaemotherapy). How ‘Inflammatory’ Cytokines may have A therapeutic Role

**DOI:** 10.1155/S0962935194000438

**Published:** 1994

**Authors:** V. Bocci

**Affiliations:** Institute of General Physiology and Nutritional Sciences University of Siena 53100 Italy

## Abstract

Immunoregulatory cytokines produced by the TH1 subset and by CD8^+^ T lymphocytes appear to brake naturally and sometimes arrest the progress of HIV infection in the early phase. It appears reasonable to assume that a mild and equilibrated stimulation of the immune system may prevent or delay the fatal transition towards the prevalent production of TH2-type cytokines. The problem is how to stimulate the immune system in a physiological fashion. In the last 7 years we have clarified the main mechanisms of action of an unorthodox immunotherapeutic method first used 40 years ago. Optimized autohaemotherapy after a brief exposure ofblood to ozone may today afford the trick of reprogramming the immune system to keep HIV at bay. The autohaemotherapeutic procedure is simple, safe, inexpensive and most likely is more effective than conventional approaches.


Mediators of Inflammation 3, 315–321 (1994)

### A reasonable approach for the treatment of HIV infection in the early phase with ozonetherapy (autohaemotherapy). How 'inflammatory' cytokines may have a therapeutic role *

V. Bocci

Institute of General Physiology and Nutritional Sciences, University of Siena, 53100 Italy.

**IMMUNOREGULATORY cytokines produced by the TH1 subset and by CD8+ T lymphocytes appear to brake naturally and sometimes arrest the progress of HIV infection in the early phase. It appears reasonable to assume that a mild and equilibrated stimulation of the immune system may prevent or delay the fatal transition towards the prevalent production of TH2-type cytokines. The problem is how to stimulate the immune system in a physiological fashion. In the last 7 years we have clarified the main mechanisms of action of an unorthodox immunotherapeutic method first used 40 years ago. Optimized autohaemotherapy after a brief exposure of blood to ozone may today afford the trick of reprogramming the immune system to keep HIV at bay. The autohaemotherapeutic procedure is simple, safe, inexpensive and most likely is more effective than conventional approaches.**

**Key words:** AIDS, Autohaemotherapy, Cytokines, Granulocyte–macrophage colony stimulating factor, HIV infection, Interferons, Interleukins, Ozone, Tumour necrosis factor-α

### Introduction

Projections of the magnitude of human immunodeficiency virus (HIV) infection by the end of the century range between 20 and 40 million worldwide but valid vaccination is not yet in sight. A number of therapeutic approaches based on the use of reverse transcriptase inhibitors, antisense oligonucleotides, proteinase inhibitors, and gene therapy are under active investigation but their final efficacy remains uncertain owing to the heterogeneity of the virus and the rapid emergence of mutants.

On the other hand, great progress has been made in the understanding of HIV pathogenesis[1–4] and recently some immunological characteristics of either long-term non-progressors or/and high-risk seronegative individuals have suggested that cell-mediated anti-HIV response, soon after infection, may be crucial for controlling the progression of the disease.[4,5] More specifically, it appears that CD8+ lymphocytes present in long-term survivors may release an as yet unidentified cytokine (momentarily denominated cell antiviral factor or CAF), heat and acid-stable, capable of blocking HIV transcription.[6,7]

There is now some consensus that besides the activity of the hypothetical CAF, a prevalent and persistent production of type-1 cytokines (supported by CD4+ TH1 cells) such as interleukin (IL)-2, 12, granulocyte–macrophage colony stimulating factor (GM–CSF) and interferon (IFN)γ[8–13] over the production of humoral immunity (supported by CD4+ TH2 cells) inducing type-2 cytokines as IL-4, 5, 6, 10 and 13[14–19] may prevent or delay the progression of HIV infection. It is noteworthy that either TH1-type or TH2-type cytokines can *in vitro*, either prevent or markedly increase, respectively, apoptosis of CD4+ cells thus, at least in part explaining the inexorable fall of CD4+ cells in blood, that has become a hallmark of acquired immunodeficiency (AIDS).[1-3,20] Moreover, for a long time it has been known that the acid-labile IFNα, present in the sera of AIDS patients, correlates with disease progression and is an inauspicious prognostic marker.[21,22] Indeed the excessive production of IFNα may be detrimental because, by enhancing MHC restricted and unspecific killing of virus infected cells, it accelerates the exhaustion of the lymphocyte pool.

At the present time, therefore, it can be concluded that in the late phase of HIV infection, an excessive production of certain cytokines such as IL-4, 6, 10, 13, IFNα, tumour necrosis factor-α (TNF),[23–25] and transforming growth factor-β (TGF-β)[26,27] favours the progressive failure of the immune system. If this assumption is correct, one should devise a therapeutic strategy aiming not only at correcting the immune dysregulation, but rather at potentiating the early attempt of the host's immune system to block or eradicate the infection.

Previous approaches based either on the exogenous administration of biological response modifiers or type-1 cytokines[9,10,28–31] have been modestly successful or palliative and this is not surprising because pharmacological administration of cyto-

*Dedicated to the memory of Giovanni Battista Rossi (deceased 20 February, 1994), who was the main force for the study of HIV infection in Italy.

© 1994 Rapid Communications of Oxford Ltd kines, as has been observed in cancer therapy, tends to dysregulate the cytokine network and to yield considerable toxicity.[32,33]

The alternative possibility put forward in this paper is to stimulate the host's early response to the virus, and try to potentiate the immune response that is apparently functioning well in long-term non-progressors. Although these subjects may be infected with a less cytopathic HIV strain than short-term survivors, it is possible that the latter, who are unfortunately the majority, have either an intrinsic defect or/and their immune system deranges more rapidly and becomes incapable of mounting an effective anti-HIV response.

This particular immune deficiency may be corrected by treating $HIV^+$ asymptomatic individuals with a fairly normal T lymphocyte $CD4^+$ level with a therapeutic procedure that, although unorthodox, has already been extensively tested in chronic viral diseases and is very simple to execute, safe, inexpensive and free of side effects.[34–39] The scope of this paper is to evaluate pros and cons of this approach that, by using the available markers for disease staging and therapy monitoring can be put to the test in clinical trials immediately. As autohaemotherapy has amply proved[37–40] to be safe and atoxic, we can undertake a phase II study directly.

### Which are the biological effects elicited by autohaemotherapy?

While ozone is the trigger, several blood components such as erythrocytes, lymphocytes, monocytes, polymorphonuclear leukocytes, platelets, and plasma components act as substrates[40] and are responsible for setting in motion a number of biological effects that, directly or indirectly, are responsible for the clinical improvement observed after the autohaemotherapeutic treatment in chronic viral diseases. (Table 1.)

**Table 1.** Relationship between blood cells and targets sensitive to effector molecules when ozone is the inducer

| Blood components | Effector molecules | Targets |
|---|---|---|
| Erythrocytes | Cytokines | T and B lymphocytes |
| | Growth hormone | Monocytes |
| Monocytes | | |
| | | Macrophages |
| | Growth inhibitors | |
| Lymphocytes | | Granulocytes |
| | Metabolites | Cytotoxic lymphocytes |
| Granulocytes | | |
| | | Natural killer cells |
| | Eicosanoids | |
| Platelets | | |
| | | Hepatocytes, |
| Plasma | | Endothelial, haematopoietic, virus infected, and neoplastic cells |

Ozone is a very reactive gas and yet, when present in dry air at 22°C, it has a half-life of about 40 min.[38] However, when it comes into contact with biological fluids, such as plasma or the lung lining fluid, it reacts immediately, particularly if the pH is below 7.4. Decomposition of ozone generates a cascade of chemical compounds such as ozonides, aldehydes, hydroxyhydroperoxides, hydrogen peroxide and lipid hydroperoxides[41] that have longer lifetimes than ozone and if unquenched, can penetrate into the cells.

Ozone has very limited specificity but preferred substrates, in decreasing order, are mono- and polyunsaturated fatty acids, cholesterol, free and protein bound cysteine, methionine, tyrosine, tryptophane, histidine and free and bound carbohydrates present in glycoproteins. All these compounds, present in cellular membranes,[42–44] in plasma and in viruses, can react with ozone and its derivatives with more or less severe alterations. However, reactions are somewhat hindered or turned off by the presence either in the plasma or in the cellular cytoplasm by an array of antioxidant compounds,[45] such as ascorbic acid, uric acid glucose, $\alpha$-tocopherol, $\beta$-carotene, lycopene, reduced glutathione and albumin. Therefore, it should be made clear that the oxidant properties of ozone on cellular and viral substrates depend upon ozone concentration and length of exposure, pH, temperature and last but not least, by the variable presence of 'scavengers' or electron donor compounds.[45]

The action of ozone derivatives on blood cells is mainly, if not exclusively, limited to their surface and, as reviewed extensively in previous articles,[40,46] results in an activation of either their metabolism or in their induction with consequent release of a number of cytokine (IFNs, ILs, haemopoietins),[47–51] growth regulatory factors, and aproteinaceous compounds such as adenosine tri-phosphate (ATP) and possibly eicosanoids (Table 1), as if the sudden and brief exposure of blood to $O_2/O_3$ switches on a great number of cellular mechanisms. Furthermore the unusual feeling of well-being noticed by most of the patients during autohaemotherapy suggests the possibility that biological effects are not limited to the stimulation of blood cells but, owing to a sudden homeostatic change, there occurs a neuroendocrinological modification, the relevance of which cannot be fully understood until we can investigate the variation of several hormonal parameters before and after the therapy. However, it must be said that the complexity of biological effects resulting from the interaction between $O_2/O_3$ on one side and the biological system on the other, are neither easily predictable, nor constantly reproducible as we are dealing with patients differing from genetic make-up, age, sex, hormonal, metabolic status, type of diet, pathology and most importantly for a variable amount of antioxidant compounds in their plasma.[49–51]

A remark is needed in regard to the virucidal effect of ozone. There is no doubt that ozone is a potent virucidal agent[52–54] that, by preferentially promoting lipid peroxidation, can inactivate more effectively lipid-enveloped viruses i.e. HIV than those with minimal lipid content. However, it is well known that in viral diseases most of the virus is located intracellularly while the free virus, except during the viraemic phase, is scarcely present in the circulation. During haemotherapy, practically only virus-infected cells present in blood can be directly exposed to $O_2$/$O_3$, while most of the virus replicates in far less accessible organs such as liver, spleen, bone marrow, lymph nodes and central nervous system. Although the postulations[55] that viral infected cells may have a decreased content of protective enzymes against oxidation still holds true, thus rendering these cells more sensitive to the oxidative effects of ozone derivatives, it remains difficult to envisage that ozonization of only a small percentage of the blood mass would be able to translate such a significant oxidative effect on the whole organism enough to reduce the virus burden. While it is true that lipid hydroperoxides have a fairly long half-life, hence theoretically they could reach any body fluid, within a few hours they are inactivated by ubiquitous chain-breaking compounds such as α-tocopherol.[45] (Bocci *et al.*, manuscript in preparation).

Moreover, even though viral infected cells present in blood are more susceptible to oxidation, they most likely survive because one of the requisites of the ozone treatment is to avoid a serious intracellular damage. Then how does ozone act?

As I purported before,[46] our working hypothesis is that ozone acts mainly by inducing blood mononuclear cells to produce immunoregulatory cytokines, particularly those produced by lymphocytes with TH1 phenotype. After incubation of ozonized blood we have shown[47–51] the release of small amounts of practically all cytokines so far tested, particularly GM-CSF, IL-2, IL-8, IFNγ, TNFα and IFNβ. After reinfusion of ozone-treated blood, mononuclear cells are supposed to home in various lymphoid (spleen, lymph nodes, thymus, Peyer's patches) and non-lymphoid (lungs, liver) organs thus priming and activating other immune cells (Fig. 1) The prolonged repetition of autohaemotherapy favours a progressively amplified activation of the immune system via numerous pathways such as activation of either major histocompatibility complex-restricted cytolysis, or nonspecific killing via activation of natural-killer cells, macrophages and neutrophils, that ultimately represents the most efficient system of destruction of viral infected cells. Although it may be a minor mechanism, the possibility cannot be excluded that the small amounts of free virus inactivated in the plasma during ozonization may act either as an endogenous immunogen or/and an activator of cell-mediated immunity. The possibility that ozone and derivatives may also inactivate either the viral gp 120, or gp 41, or the cellular receptor(s) (CD4 and CD26 for HIV) remains to be demonstrated as ozone acts preferentially on phospholipids. The activation of metabolism and possibly psychosomatic factors may play ancillary roles.

### Some advice regarding the most suitable administration of ozone and of the optimized procedure

This topic is important because ozone has in my opinion, a low therapeutic index and we must strive to achieve an effective immunostimulation with the least cellular toxicity. It suffices here to say that during the last 4 years we have extensively evaluated the toxic effects by measuring the level of ozone induced haemolysis (below 3.5%),[49–51] possible formation of metahaemoglobin (always absent), morphologic damage (absent below 80 μg/ml of ozone) as evaluated by electron microscopy, plasma levels of lipid hydroperoxides (increasing 3-fold after ozonization with 90 μg/ml ozone/ml of blood but rapidly returning to baseline values), and intraerythrocytic reduced glutathione levels (never below 10% and rapidly restored).[50] Unexpectedly and contrary to the ozone dosage usually used in autohaemotherapy (from 5 to 40 μg/ml $O_3$ per ml of blood), we have found that we could raise the ozone levels, and the most effective concentrations without toxicity range between 50 and 80 μg/ml depending upon the individual plasma levels of anti-oxidant

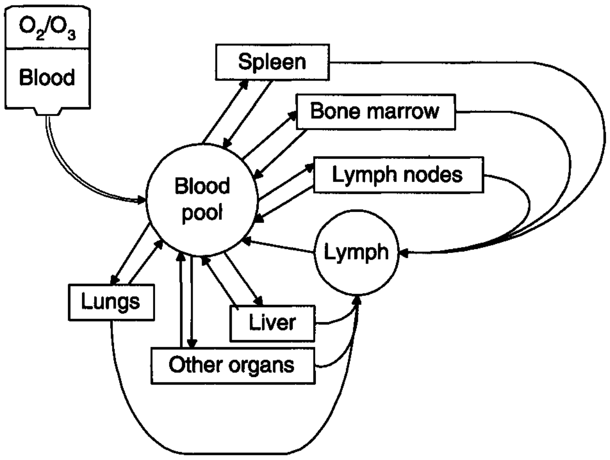

FIG. 1. The scheme suggests that blood mononuclear cells, after reinfusion, leave the blood compartment, home in various lymphoid and non-lymphoid organs and release cytokines finally responsible for the activation of the immune system. Activated erythrocytes improve oxygenation and metabolism.

compounds. The concept[46,49] of correlating the production of cytokines by blood mononuclear cells *vs.* ozone concentration has represented a crucial advantage and it has become an indispensable end-point. Today it has become unthinkable to assess the correctness of the ozone concentrations by bubbling the gas through the blood sample, as has been done recently.[56]

On the other hand it appears evident that the exposure of a precise volume of blood to a known volume of ozone with a predetermined concentration and for a definite time allows a reliable stoichiometric procedure. For each autohaemotherapeutic treatment we advise collecting 250–300 ml of blood in bags containing 25 IU/ml of calciheparin/ml of blood plus 5 mM $Ca^{2+}$. The elimination of the commonly used anticoagulant solution of citrate–phosphate–dextrose (CPD) and the addition of 5 mM $Ca^{2+}$ markedly enhances the production of cytokines as reported in detail elsewhere.[49,51] This point is emphasized because in viral infections it is necessary to activate the cellular immune response with the aim of eradicating or at least slowing the progress of the disease.

The ozone generator is reliable and ozone concentration can be precisely checked by photometric analysis. The $O_2/O_3$ gas mixture is simply and rapidly added with a syringe to reach a final concentration of 70 μg/ml ozone per ml of blood. A modest hyperbaric pressure occurs as commercially available bags of 450 ml may finally contain 300 ml of blood and 240 ml of gas mixture. In terms of cytokine induction, an ozone concentration below 40 μg/ml is modestly effective and our recent experience has taught us that, depending upon the variable presence of antioxidants in the plasma, a range of ozone concentration between 50 and 80 μg/ml gives reliable results and currently we use 70 μg/ml/per ml of blood.

Immediately after the gas addition, the bag is gently mixed up and down by hand for up to 5 min. After carrying out analysis of the $pO_2$, $pCO_2$ and pH every minute, we have noted that within 3–5 minutes the $pO_2$ tension reaches a plateau. The fact that ozone is more soluble than oxygen suggests no further need of mixing and transfusion in the donor is carried out during the following 20–30 min with occasional mixing to minimize erythrocyte sedimentation. The speed of reinfusion is checked by a common set for blood transfusion. The procedure is therefore simple, without needing any technical device so that it could be easily carried out even in poorly equipped hospitals in third-world countries.

The frequency of treatments is fairly flexible but the preferred schedule is thrice weekly for the first two months and twice weekly for the following four months. Even if a total blood volume of about 17 l is exposed to ozone during this period, it corresponds to only about 7% of the lymphocyte mass.[40,57] If it is beneficial, the treatment can be continued once a week, or every other week, indefinitely.

Autohaemotherapy does not procure side effects and often patients report a sense of well-being and euphoria. In comparison to the toxic syndrome noted after administration of potent cytokine inducers,[58,59] reasons for the lack of toxicity after autohaemotherapy discussed at length elsewhere[40] can be attributed to the physiological-like release of cytokines[60] in various cellular microenvironments after the homing of the reinfused cells. The scheme reported in Fig. 1 illustrates how activated immune cells may circulate without any substantial increase of cytokine levels in the circulation which is the main cause of toxicity after pharmacological administration.[32,33]

### Conceptual and technical disadvantages of this approach

First, although the activation *ex vivo* of blood mononuclear cells with ozone is very mild, the possibility cannot be excluded that some cytokines such as TNFα and GM–CSF may reactivate the replication of a latent HIV infection.[61,62] Furthermore, particularly in the first phase of the treatment, increased oxidative stress in HIV positive subjects and the killing of infected cells may also favour the reactivation of the disease. This possibility is clearly serious and must be controlled (at least in the initial phase) by a concomitant administration of zidovudine, *N*-acetyl-L-cysteine and by strictly monitoring cellular, immunological and virological markers as well as the clinical status for eventually interrupting the treatment. In order to stabilize the plasma levels of antioxidants and to make sure that patients receive a normal vitaminic support we have always prescribed a daily multivitamin (including vitamin C and E) supplement.

Subjects hypersensitive to heparin or under anticoagulant therapy, or aspirin, or prone to the haemorrhagic syndrome, or thrombocytopenia have to be carefully evaluated before inclusion. Other criteria for exclusion do not need to be discussed here. If venous access is very difficult, alternative possibilities are discussed in the following section.

### Are other routes of administration of ozone equally useful?

Mattassi[36] has extensively reviewed this issue and one is amazed by the number of administration routes (Table 2) and the relatively low toxicity most likely inhibited by the body anti-oxidant reservoir.[45] Unfortunately, therapeutic efficacy has been evaluated in most cases in anecdotic fashion and for these various routes it appears impossible to define a

**Table 2.** Routes for the administration of ozone

| Parenteral | Local insufflation |
|---|---|
| Intravenous | Nasal |
| Intra-arterial | Tubal |
| Intramuscular | Oral |
| Subcutaneous | Vaginal |
| Intra-articular | Vesical |
| | Rectal |
| | Cutaneous |

stoichiometric relationship as there is between a precise volume of blood and ozone. In fact, even by controlling the volume of gas infused, the volume of blood flowing in a vessel or being exposed in an internal cavity or tissue, remains unknown. Thus, the first drawback is that ozone dosage has to be defined on a trial and error basis.

In spite of a slow and controlled administration, and of $O_2/O_3$ solubility and capillary absorption, the intra-arterial and particularly intravenous routes can be painful and predispose to pulmonary embolism. When up to a total of 120 ml of gas have been intravenously injected daily, ozone may give rise to a transient toxic syndrome. Administration of a fairly large volume of ozone (up to 100 ml) via intramuscular and subcutaneous routes is usually painful and therapeutic efficacy remains unclear. Since last year, as an alternative to these routes and autohaemotherapy, we have been evaluating the infusion of isotonic saline (300 ml) contained in a 500 ml glass bottle saturated with 360 ml of $O_2/O_3$ (38 mg of ozone). Even patients with precarious venous access show a good compliance and so far, no untoward effects have been noted.

Fahmy[63] has reported that intra-articular administration of ozone associated or not to autohaemotherapy can be beneficial in rheumatoid arthritis. This is surprising because at the site of the rheumatoid lesion there is an abnormal release of inflammatory cytokines and active radicals, and one would expect that injection of ozone would worsen the disease. Thus, the paradoxic effect of ozone could be tentatively explained by either the enhanced release of cytokine antagonists or of cytokines such as IL-10 and TGF$\beta$1 able to suppress autoreactive cytotoxic T cells, or by ablating the production of autoantigens, or by inducing the expression of superoxide dismutase.

Of the various local treatments, the cutaneous one (for torpid ulcers or necrotic lesions due to chronic limb ischaemia), when properly done, is free of side effects and effective especially when combined to autohaemotherapy.[64]

Rectal insufflation of up to 800 ml of $O_2/O_3$ has been practised with beneficial effects in AIDS patients with intractable diarrhoea.[65] The same route was evaluated by Knoch *et al.*[66] showing significant metabolic modifications as measured by haemogas (higher $pO_2$ than normal) and biochemical analysis. These results imply conspicuous absorption of $O_2/O_3$ through the mucosal lining with consequent effects on intestinal capillary blood. Usually, insufflation of about 300 ml of $O_2/O_3$ with an ozone concentration of 20 μg/ml (i.e. a total of 6 mg of ozone), 2–3 times weekly, is well tolerated and seems effective in the treatment of chronic hepatitis and may be useful in HIV infected patients without venous access. However, in comparison to haemotherapy, unless we define some markers, it appears difficult to establish an optimal dosage for this route. Nonetheless, it would be worthwhile to clarify whether there is some ozone absorption via intestinal lymphatics with the possibility of either activating mononuclear cells in intestinal lymph and in the vast retroperitoneal lymph node network. Ozone may subtly alter mucosal permeability thus enhancing absorption via lymphatics of compounds derived from the bacterial flora with immunoenhancing properties. With regard to this, insufflation of $O_2/O_3$ in the peritoneal cavity may also offer some advantages although this route is not as practical as the rectal one.

### Concluding remarks

HIV infection poses a challenging medical problem with no ready solution in sight. However, the observation that a few patients can survive in an apparently healthy state for over 10 years after HIV infection has provided an important clue for tackling the problem. As a matter of fact it is well known that, during epidemics, a variable percentage of humans can resist and even overcome the most virulent viral infection, depending upon the efficiency of their immune system. This certainly depends upon a number of genetic, metabolic, hormonal and nutritional factors but there are no fool-proof markers to

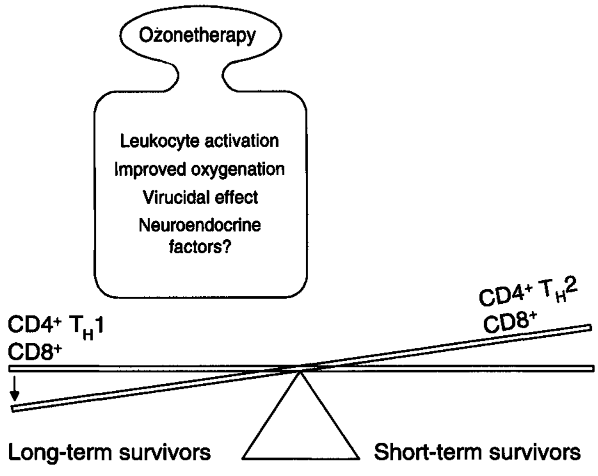

FIG. 2. The scheme indicates the dynamic situation that may lead either to AIDS or to an apparent disease-free state. The balance may tilt towards long survival by using autohaemotherapy after exposing autologous blood to $O_2/O_3$.

predict the individual resistance to the infection. A limitation of the proposed approach is to select HIV infected people that are likely to benefit from the therapy and this will impose strict eligibility criteria. With regard to selection of potentially responsive patients, a meaningful approach based on the evaluation of TH function has been proposed by Clerici and Shearer[5,13] and may be useful. As shown in Fig. 2, the aim is to modify the natural course of the disease by activating cellular immune reactivity, thus creating the condition for a longer survival.

An obvious and pressing question is what to do when patients have progressed to AIDS. While the approach proposed here may well be useless or even detrimental because it may upregulate HIV replication, one has to be extremely cautious in attempting allohaemotherapy because ozone-activated, allogenic lymphocytes may cause graft *vs.* host disease.

At present, therapy with biological response modifiers has not been rewarding but nonetheless several approaches are being evaluated.[67,68] In spite of several observations[69–72] of suppression of HIV replication in blood mononuclear cells *in vitro*, IFNα and β, used as single agents, are minimally useful in HIV infected patients,[30] except in Kaposi's sarcoma.[28,29] Administration of polyethylene/glycol modified IL-2 may improve $CD4^+$ cell counts[31] but it procures a certain level of toxicity and may perturb the cytokine network with an unpredictable outcome. Gene therapy, after cloning the IL-2 and IFNγ genes in lymphocytes, offers the alternative possibility of an endogenous, physiological-like process but, besides the technical problem and the cost of a personalized therapy, there remains the problem of getting an optimal and stable therapeutic level. Administration of haematopoietic growth factors may be useful but it needs to be optimized and made safe regarding modulation of HIV replication.[73]

Thus the procedure of autohaemotherapy appears advantageous because it is simple to perform and potentially effective because it involves an equilibrated release of type 1-like cytokines. In comparison to potent mitogens, ozone induces a small production of cytokines that, owing to a physiological distribution, does not procure toxicity Although it cannot be measured, ozone may also induce the production of CAF or other factors involved in blocking HIV infection. Moreover the procedure is safe and so inexpensive to be afforded by third-world countries.

Autohaemotherapy was first introduced by Wehrly[34] in 1954 but unfortunately it has been used in an empirical fashion and without knowing the relevant mechanism of action. By mere coincidence, I stumbled upon this problem and I hope to have contributed 'to teach an old method new tricks' that may be exploited for restoring human health.

### References

1. Fauci AS. The human immunodeficiency virus: infectivity and mechanisms of pathogenesis. *Science* 1988; **239**: 617–622.
2. Pantaleo G, Graziosi C, Fauci AS. The immunopathogenesis of human immunodeficiency virus infection. *N Engl J Med* 1993; **328**: 327–335.
3. Weiss RA. How does HIV cause AIDS? *Science* 1993; **260**: 1273–1279.
4. Levy JA. HIV pathogenesis and long-term survival. *AIDS* 1993; **7**: 1401–1410.
5. Clerici M, Shearer GM. A $T_H1 \rightarrow T_H2$ switch is a critical step in the etiology of HIV infection. *Immunol Today* 1993; **14**: 107–111.
6. Walker CM, Levy JA. A diffusible lymphokine produced by $CD8^+$ T lymphocytes suppresses HIV replication. *Immunology* 1989; **66**: 628–630.
7. Brinchmann JE, Gaudernack G, Vartdal F. $CD8^+$ T cells inhibit HIV replication in naturally infected $CD4^+$ T cells. Evidence for a soluble inhibitor. *J Immunol* 1990; **144**: 2961–2966.
8. Murray HW, Rubin BY, Masur H, Roberts RB. Impaired production of lymphokines and immune (gamma) interferon in the acquired immunodeficiency syndrome. *N Engl J Med* 1984; **310**: 883–889.
9. Siegel JP, Clifford Lane H, Stocks NI, Quinnan GV, Jr., Fauci AS. Pharmacokinetics of lymphocyte-derived and recombinant DNA-derived interleukin-2 after intravenous administration to patients with the acquired immunodeficiency syndrome. *J Biol Resp Mod* 1985; **4**: 596–601.
10. Murphy PM, Clifford Lane H, Gallin JI, Fauci AS. Marked disparity in incidence of bacterial infections in patients with the acquired immunodeficiency syndrome receiving interleukin-2 or interferon-gamma. *Ann Intern Med* 1988; **108**: 36–41.
11. Lewis DB, Wilson CB. Gamma-interferon: an immunoregulatory lymphokine with immunotherapeutic potential. *Pediatr Infect Dis J* 1990; **9**: 642–651.
12. Kiniwa M, Gately M, Gubler U, Chizzonite R, Fargeas C, Delespesse G. Recombinant interleukin-12 suppresses the synthesis of immunoglobulin E by interleukin-4 stimulated human lymphocytes. *J Clin Invest* 1992; **90**: 262–266.
13. Clerici M, Lucey DR, Berzofsky JA, *et al.* Restoration of HIV-specific cell-mediated immune responses by interleukin-12 *in vitro*. *Science* 1993; **262**: 1721–1724.
14. Finkelman FD, Katona IM, Urban JF, Jr., *et al.* IL-4 is required to generate and sustain *in vivo* IgE responses. *J Immunol* 1988; **141**: 2335–2341.
15. Tosato G, Seamon KB, Goldman ND, *et al.* Monocyte-derived human B-cell growth factor identified as interferon-$\beta_2$ (BSF-2, IL-6). *Science* 1988; **239**: 502–504.
16. D'Andrea A, Aste-Amezaga M, Valiante NM, Ma X, Kubin M, Trinchieri G. Interleukin 10 (IL-10) inhibits human lymphocyte interferon gamma-production by suppressing natural killer cell stimulatory factor/IL-12 synthesis in accessory cells. *J Exp Med* 1993; **178**: 1041–1048.
17. Cassatella MA, Meda L, Bonora S, Ceska M, Constantin G. Interleukin 10 (IL-10) inhibits the release of proinflammatory cytokines from human polymorphonuclear leukocytes. Evidence for an autocrine role of tumor necrosis factor and IL-1β in mediating the production of IL-8 triggered by lipopolysaccharide. *J Exp Med* 1993; **178**: 2207–2211.
18. Del Prete G, De Carli M, Almerigogna F, Giudizi MG, Biagiotti R, Romagnani S. Human IL-10 is produced by both type 1 helper (Th1) and type 2 helper (Th2) T cell clones and inhibits their antigen-specific proliferation and cytokine production. *J Immunol* 1993; **150**: 353–360.
19. de Waal Malefyt R, Figdor CG, Huijbens R, *et al.* Effects of IL-13 on phenotype, cytokine production, and cytotoxic function of human monocytes. *J Immunol* 1993; **151**: 6370–6381.
20. Redfield RR, Burke DS. HIV infection: the clinical picture. *Sci Amer* 1988; **259**: 70–78.
21. De Stefano E, Friedman RM, Friedman-Kien AE, *et al.* Acid-labile human leucocyte interferon in homosexual man with Kaposi's sarcoma and lymphadenopathy. *J Infec Dis* 1982; **146**: 451–455.
22. Capobianchi MR, Mattana P, Dianzani F. Potentiation of interferon-α *in vitro* antiviral activity by interferon-gamma is not abrogated by antibody to interferon-gamma. *J Interferon Res* 1993; **13**: 53–55.
23. Molina JM, Scadden DT, Byrn R, Dinarello CA, Groopman JE. Production of tumor necrosis factor α and interleukin 1β by monocytic cells infected with human immunodeficiency virus. *J Clin Invest* 1989; **84**: 733–737.
24. Poli G, Kinter A, Justement JS, *et al.* Tumor necrosis factor α functions in an autocrine manner in the induction of human immunodeficiency virus expression. *Proc Natl Acad Sci USA* 1990; **87**: 782–785.
25. Lähdevirta J, Maury CPJ, Teppo AM, Repo H. Elevated levels of circulating cachectin/tumor necrosis factor in patients with acquired immunodeficiency syndrome. *Amer J Med* 1988; **85**: 289–291.
26. Kekow J, Wachsman W, McCutchan JA, *et al.* Transforming growth factor-β and suppression of humoral immune responses in HIV infection. *J Clin Invest* 1991; **87**: 1010–1016.
27. Kehrl JH, Taylor A, Kim SJ, Fauci AS. Transforming growth factor-β is a potent negative regulator of human lymphocytes. In: Anagnostou A, Dainiak N, Najman A, eds. *Negative regulators of hematopoiesis. Studies on their nature, action, and potential role in cancer therapy.* New York: Annals New York Academy of Sciences, 1991; 345–353.
28. Rios A, Mansell PWA, Newell GR, Reuben JM, Hersh EM, Guttermann JU. Treatment of acquired immunodeficiency syndrome-related Kaposi's sarcoma with lymphoblastoid interferon. *J Clin Oncol* 1985; **3**: 506–512.
29. Groopman JE, Gottlieb MS, Goodman J, *et al.* Recombinant alpha-2 interferon therapy for Kaposi's sarcoma associated with the acquired immunodeficiency syndrome. *Ann Intern Med* 1984; **100**: 671–676.
30. Clifford Lane H. The role of α-interferon in patients with human immunodeficiency virus infection. *Semin Oncol* 1991; **18**: 46–52.

31. Wood R, Montoya JG, Kundu SK, Schwartz DH, Merigan TC. Safety and efficacy of polyethylene glycol-modified interleukin-2 and zidovudine in human immunodeficiency virus type 1 infection: a phase I/II study. *J Infec Dis* 1993; **167**: 519–525.
32. Lotze MT, Matory YL, Rayner AA, *et al.* Clinical effects and toxicity of interleukin-2 in patients with cancer. *Cancer* 1986; **58**: 2764–2772.
33. Bocci V. Tumor therapy with biological response modifiers. Why is progress slow? *EOS—J Immunol Immunopharmacol* 1990; **X**: 79–82.
34. Wehrli F, Steinbart H. Erfahrungen mit der haematogenen oxydations—therapie (HOT). *Ars Medici* 1954; **10**: 44–51.
35. Rokitansky O. Klinik und biochemie der ozontherapie. *Hospitals* 1982; **52**: 643–647.
36. Mattassi R. *Ozonoterapia*. Milano:Organizzazione Editoriale Medico Scientifica, 1985; 1–179.
37. Jacobs M. Th-Untersusuchung uber zwischerfdlle und typische komplikationen in der ozon. Sauerstoff therapie. *Ozonachrichten* 1986; **5**: 1–5.
38. Rilling S, Viebahn R. *The use of ozone in medicine*. Heidelberg:Haug K.F. Publ., 1987; 1–187.
39. Konrad H. Ozone therapy for viral diseases. In: *Proceedings 10th Ozone World Congress 19–21 March 1991, Monaco*. Zurich: International Ozone Association, 1991; 75–83.
40. Bocci V. Autohaemotherapy after treatment of blood with ozone. A reappraisal. *Int Med Res* 1994; **22**. (In press)
41. Prior WA. Ozone in all its reactive splendor. *J Lab Clin Med* 1993; **122**: 483–486.
42. Goldstein BD, Balchum OJ. Effect of ozone on lipid peroxidation in the red blood cell. *Proc Soc Exp Biol Med* 1967; **126**: 356–359.
43. Buckley RD, Hackney JD, Clark K, Posin C. Ozone and human blood. *Arch Environ Health* 1975; **30**: 40–43.
44. Freeman BA, Mudd JB. Reaction of ozone with sulfhydryls of human erythrocytes. *Arch Biochem Biophys* 1981; **208**: 212–220.
45. Halliwell B, Gutteridge JMC. The antioxidants of human extracellular fluids. *Arch Biochem Biophys* 1990; **280**: 1–8.
46. Bocci V. Ozonization of blood for the therapy of viral diseases and immunodeficiences. A hypothesis. *Med Hypotheses* 1992; **39**: 30–34.
47. Bocci V, Paulesu L. Studies on the biological effects of ozone 1. Induction of interferon gamma on human leucocytes. *Haematologica* 1990; **75**: 510–515.
48. Paulesu L, Luzzi E, Bocci V. Studies on the biological effects of ozone: 2. Induction of tumor necrosis factor (TNF-α) on human leucocytes. *Lymphokine Cytokine Res* 1991; **10**: 409–412.
49. Bocci V, Luzzi E, Corradeschi F, Paulesu L, Di Stefano A. Studies on the biological effects of ozone. 3. An attempt to define conditions for optimal induction of cytokines. *Lymphokine Cytokine Res* 1993; **12**: 121–126.
50. Bocci V, Luzzi E, Corradeschi F, *et al.* Studies on the biological effects of ozone. 4. Cytokine production and glutathione levels in human erythrocytes. *J Biol Regul Homeost Agents* 1993; **7**: 133–138.
51. Bocci V, Luzzi E, Corradeschi F, Paulesu L. Studies on the biological effects of ozone. 5. Evaluation of immunological parameters and tolerability in normal volunteers receiving ambulatory autohaemotherapy. *Biotherapy* 1994; **7**: 1–10.
52. Akey D, Walton TE. Liquid-phase study of ozone inactivation of Venezuelan Equine Encephalomyelitis virus. *Appl Environ Microbiol* 1985; **50**: 882–886.
53. Carpendale MTF, Freeberg JK. Ozone inactivates HIV at noncytotoxic concentrations. *Antivir Res* 1991; **16**: 281–292.
54. Wells KH, Latino J, Gavalchin J, Poiesz BJ. Inactivation of human immunodeficiency virus type 1 by ozone *in vitro*. *Blood* 1991; **78**: 1882–1890.
55. Bocci V. May hyperbaric oxygenation be useful to patients with AIDS? *J Biol Regul Homeost Agents* 1987; **1**: 201.
56. Garber GE, Cameron DW, Hawley-Foss N, Greenway D, Shannon ME. The use of ozone-treated blood in the therapy of HIV infection and immune disease: a pilot study of safety and efficacy. *AIDS* 1991; **5**: 981–984.
57. Westermann J, Pabst R. Distribution of lymphocyte subsets and natural killer cells in the human body. *Clin Investig* 1992; **70**: 539–544.
58. Levine AS, Sivulich M, Wiernik PH, Levy HB. Initial clinical trials in cancer patients of polyriboinosinic-polyribocytidylic acid stabilized with poly-L-lysine, in carboxymethylcellulose [poly(ICLC)], a highly effective interferon inducer. *Cancer Res* 1979; **39**: 1645–1650.
59. Mackensen A, Galanos C, Engelhardt R. Treatment of cancer patients with endotoxin induces release of endogenous cytokines. *Pathobiology* 1991; **59**: 264–267.
60. Bocci V. Roles of interferon produced in physiological conditions. A speculative review. *Immunology* 1988; **64**: 1–9.
61. Mellors JW, Griffith BP, Ortiz MA, Landry ML, Ryan JL. Tumor necrosis factor-α/cachectin enhances human immunodeficiency virus type 1 replication in primary human macrophages. *J Infec Dis* 1991; **163**: 78–82.
62. Perno CF, Yarchoan R, Cooney DA, et al. Replication of human immunodeficiency virus in monocytes: granulocyte/macrophage colony-stimulating factor (GM-CSF) potentiates viral production yet enhances the antiviral effect mediated by 3′-azido-2′3′-dideoxythymidine (AZT) and other dideoxynucleoside congeners of thymidine. *J Exp Med* 1989; **169**: 933–951.
63. Fahmy Z. Influence of ozone therapy in rheumatoid arthritis. In: *Proceedings 10th Ozone World Congress, 19–21 March 1991, Monaco, Vol 3*. Zurich: International Ozone Association, 1991; 85.
64. Werkmeister H. The efficacy of $O_2/O_3$ low-pressure application in badly healing wounds. In: *Proceedings 10th Ozone World Congress 19-21 March 1991, Monaco*. Zurich: International Ozone Association, 1991; 41–53.
65. Carpendale MT, Freeberg J, Mcleod Griffiss J. Does ozone alleviate AIDS diarrhea? *J Clin Gastroenterol* 1993; **17**: 142–145.
66. Knoch HG, Roschke W, Klug W. Ozone/oxygen therapy in proctology. *Ozonachrichten* 1987; **6**: 51–70.
67. Johnston MI, Hoth DF. Present status and future prospects for HIV therapies. *Science* 1993; **260**: 1286–1293.
68. Yarchoan R, Mitsuya H, Broder S. Challenges in the therapy of HIV infection. *Immunol Today* 1993; **14**: 303–309.
69. Yamada O, Hattori N, Kurimura T, Kita M, Kishida T. Inhibition of growth of HIV by human natural interferon *in vitro*. *AIDS Res Hum Retrovirus* 1988; **4**: 287–294.
70. Bednarik DP, Mosca JD, Raj NBK, Pitha PM. Inhibition of human immunodeficiency virus (HIV) replication by HIV-trans-activated $\alpha_2$-interferon. *Proc Natl Acad Sci USA* 1989; **86**: 4958–4962.
71. Michaelis B, Levy JA. HIV replication can be blocked by recombinant human interferon beta. *AIDS* 1989; **3**: 27–31.
72. Bourinbaiar AS, Nagomy R. Inhibitory effect of natural interferon alpha on human immunodeficiency virus type 1 transmission from epithelial cells to lymphocytes *in vitro*. *Eur J Pharmacol* 1993; **230**: 15–22.
73. Groopman JE, Feder D. Hematopoietic growth factors in AIDS. *Semin Oncol* 1992; **19**: 408–414.

ACKNOWLEDGEMENTS. I am very grateful to Mrs Helen Carter Bocci for English revision and to Miss Patrizia Marrocchesi for her skill and patience in preparing the manuscript. This work has been partially supported by MURST (40%) and by the National Research Council (CNR), Rome—Target Project 'Applicazioni cliniche della ricerca oncologica'.

**Received 2 June 1994; accepted 9 June 1994**
